# Traditional Cigarette and Poly-Tobacco Use Are Associated with Medical Opioid Use in Rural Areas of the US

**DOI:** 10.3390/ijerph182211864

**Published:** 2021-11-12

**Authors:** Mariaelena Gonzalez, Ashley Sanders-Jackson

**Affiliations:** 1 Department of Public Health, School of Social Sciences Humanities and the Arts, University of California Merced, Merced, CA 95343, USA; 2 Nicotine and Cannabis Policy Center, University of California Merced, Merced, CA 95343, USA; 3 Department of Advertising and Public Relations, College of Communications Arts and Sciences, Michigan State University, East Lansing, MI 48824, USA; sande411@msu.edu

**Keywords:** tobacco use, opioids, rural populations, smoking

## Abstract

Introduction: Medical prescriptions for opioids are higher in rural areas of the US as compared to urban areas. Tobacco use may also play a role in this process. This analysis examines the association between differing types of tobacco use and medical opioid use. Methods: We analyze the relationship between tobacco product use and medical opioid use among the US general population living in rural (non-metropolitan) areas using the publicly available sample adult file 2019 National Health Interview Survey (NHIS) (*n* = 5028). Tobacco use was classified into the following categories: only using cigarettes, only using e-cigarettes/vapes, only using cigars, only using smokeless tobacco, or using two or more of the following products. We used a binary logistic regression, controlling for individual differences. Results: Individuals who reported using only traditional cigarettes (and no other tobacco product, OR = 1.62, 95% CI: 1.31, 2.01), or who reported being a poly-tobacco users (OR = 2.13, 95% CI: 1.40, 3.22) had higher odds of medical opioid use in the last twelve months. Conclusion: Results suggest a link between tobacco use, particularly cigarette use and poly-tobacco use, and medical opioid use in rural communities. Clinical and structural level interventions need to be implemented in rural communities to reduce comorbid tobacco and opioid use.

## 1. Introduction

Death and injury from non-medical prescription opioid misuse is one of the contributing factors to a higher unintentional injury death rate in rural regions of the United States [1,2]. Medical prescriptions for opioids are higher in rural areas of the US as compared to urban areas [3]. This is important because there is a link between medical opioid use and the use of illegal narcotics and non-medical opioids [4,5]. The ratio of non-medical prescription opioid users to medical users is higher in rural regions as well [6], suggesting this is an important area for public health research to address the opioid epidemic [7,8,9].

Tobacco use may also play a role in this process. The co-use of tobacco and opioids is extremely high among individuals with substance use disorder in comparison to other substances that might be co-used [10,11]. Tobacco use has been associated with a greater experience of physical pain [12], and tobacco users may be at a greater risk for requiring prescribed opioids [13]. Therefore smoking may be a risk factor for opioid use among individuals suffering from chronic pain [14,15].

While there is a large literature examining the relationship between tobacco and non-medical opioid use, few studies examine the relationship between medical use and tobacco in rural regions. This study tests if the use of various tobacco products is associated with medical opioid use among US adults in rural areas.

## 2. Methods

We analyze the relationship between tobacco product use and medical opioid use among the US general population living in rural (non-metropolitan) areas using the publicly available sample adult file 2019 National Health Interview Survey (NHIS) (*n* = 5028). The NHIS is a cross-sectional, national survey of households in the United States (US) conducted annually, and is the primary source of health statistics in the US. The total household response rate in 2019 was 61.1% [16]. Because we used the publicly available 2019 NHIS files, this research is not considered as human subject research.

### 2.1. Dependent Variable

Medical opioid use was defined as using an opioid pain reliever which had been prescribed by a health professional sometime during the last 12 months.

### 2.2. Independent Variables

Our primary variable of interest was tobacco use, which was classified into the following categories: only using cigarettes, only using e-cigarettes/vapes, only using cigars, only using smokeless tobacco, or using 2 or more of the following products—traditional cigarettes, e-cigarettes/vapes, pipes, cigars, or smokeless tobacco. A total of 14 individuals who reported only using pipes were excluded from the analysis due to the small size of this group.

We included the following individual difference measures as co-variates—age (18–34 years of age, 35–64, 65 or older), sex (male/female), race/ethnicity (non-Hispanic (NH) White, NH African American, NH Asian, Latino, NH Other), education (not graduated from high school, High School degree or GED, some college, bachelor’s or higher), income to federal poverty level ratio (under 1, 1–1.99, 2–3.99, 4 or higher) and US region (west, northeast, Midwest, south).

### 2.3. Analysis

We used a multivariable binary logistic regression to test the association between tobacco product use and medical opioid use, controlling for sex, age, race/ethnicity, education, income to federal poverty level ratio, and US region. In order to account for the complex survey design of the NHIS, data were weighted according to directions provided by the US National Center for Health Statistics in the NHIS documentation [16]. Stata version 15 was used for the analysis.

## 3. Results

The majority of respondents (72.81%, 95% CI: 71.12%, 74.44%, Table 1) did not use tobacco products, and using traditional cigarettes (15.32%, 95% CI: 13.94%, 16.80%) was the most popular form of tobacco use. Respondents were generally NH White (80.63%, 95% CI: 75.52%, 84.89%), and 35–64 years of age (48.92%, 95% CI: 47.48%, 50.37%). Only 17.14% (95% CI: 15.37%, 19.06%) of the sample reported a college education or higher—around a third of respondents reported obtaining a high school GED (34.26%, 95% CI: 32.37%, 36.20%) or some college (32.45%, 95% CI: 30.45%, 34.52%) education.

### 3.1. Traditional Cigarette Users and Poly-Tobacco Users Have Higher Odds of Medical Opioid Use

Individuals who reported using only traditional cigarettes (and no other tobacco product, OR = 1.62, 95% CI: 1.31, 2.01, Table 2), or who reported being poly-tobacco users (OR = 2.13, 95% CI: 1.40, 3.22) had higher odds of medical opioid use in the last 12 months. Using only e-cigarettes/vapes, cigars, or smokeless tobacco was not associated with prescribed opioid use in the past 12 months.

### 3.2. Latinos and Young Adults Have Lower Odds of Medical Opioid Use, Women and Low-SES Individuals Have Higher Odds

Latinos (OR = 0.53, 95% CI: 0.32, 0.86) and young adults (OR = 0.54, CI: 0.40, 0.72) had lower odds of medical opioid use and individuals who identified as female (OR = 1.91 95% CI: 1.55, 2.34) or were below the Federal Poverty Line (OR = 1.62, 95% CI: 1.18, 2.23) had a greater risk medical opioid use in the last 12 months. 

## 4. Discussion

Previous research shows that opioid overdose rates are higher in rural areas [17] and there may be a link between tobacco and opioid use. There are a number of factors that put individuals living in rural areas at risk for greater tobacco use. Rural areas have higher rates of tobacco use, and individuals in these areas smoke more cigarettes per day than urban smokers, rural areas have been the focus of tobacco industry marketing, often have less protective tobacco-control policies, and smoking rates are higher in rural areas of the US, compared to urban areas [18,19,20,21]. Given that there is a link between prescribed opioid use and the use of illegal narcotics and that the discontinuation of opioids prescribed by a health professional can lead to use of non-prescription opioid use [13,22], understanding this relationship in the context of tobacco is important. Our results suggest that there is a link between tobacco use, particularly cigarette use and poly-tobacco use, and medical opioid use in rural communities. Taken together, these facts suggest that addressing the link between smoking and opioid use may be an important factor in reducing the misuse of opioids in these regions of the US.

Additionally, women and lower-income individuals may be at particularly high risk for medical use of opioids. Lower-income individuals face a variety of structural factors, such as workplace environments that require repetitive motion and high levels of stress, that may be associated with a greater experience of physical pain [23]. Women often describe more pain and thus may be at greater risk for opioid use [24]. This may need be considered in intervention design such that interventions should be tailored to these populations or at least considering these populations in their design processes. While this analysis does not examine why or why not individuals are prescribed opioids, the fact that Latinos had lower odds of medical opioid use may reflect the fact that this group is at high risk of being uninsured. Young adults may be at lower odds of medical opioid use due to the fact they are more likely to receive short-term opioid prescription from dentists for tooth extraction [25]. There are two levels of intervention that are easily identifiable: (1) clinical interventions and (2) population-level health interventions. Currently, due to high rates of co-use between tobacco and opioids, co-treatment as a standard of care may be helpful in a clinical context [11]. While the direction of the association of tobacco and medical opioid use cannot be inferred from cross sectional data, it is possible that currently existing interventions for reducing tobacco use could help reduce opioid use. If so, there are steps rural health care systems and providers can take to reduce tobacco use in the hope of reducing opioid use. Encouraging smoking cessation through validated approaches may be an important first step as well as creating effective anti-smoking interventions both through the medical system and media that are culturally tailored to rural participants. Currently, due to high rates of co-use between tobacco and opioids, co-treatment as a standard of care may be helpful [11]. Second, population-level health interventions, such as stricter policies on tobacco use, anti-smoking campaigns, clean indoor air laws, harm reduction campaigns to support reduced use of opioids [26] and other strategies may be helpful.

## 5. Limitations

The information in the survey is provided through self-reporting, and suffers from the limitations of this type of data. These data are cross-sectional in nature. Though our results suggest a link between tobacco use and prescribed opioid use in rural communities, we are not able to establish causality. Future research should be longitudinal in nature. Though we control for a number of individual differences, more granular analysis considering subgroups (e.g., separate analyses for rural men and women) might provide additional direction for how tobacco use is linked to opioid use in different subgroups. There is an epidemic of opioid use in the United States which is understood to be a complex and multifaceted epidemic that includes poor industry behavior and underlying social conditions, such as poverty, that impact individual behavior [7,8,9]. Due to the complex and multifaceted nature of this problem, including additional important variables, such as proximity to a hospital, coverage by tobacco control policy, county level poverty, etc., may help disentangle how much of the detected relationship is based on environmental factors that cause both the need for prescription opioid use and tobacco use. Further, additional research needs to be completed to disentangle the relationship between tobacco use and physical pain when controlling for possible covariates.

## 6. Conclusions

Individuals who are in chronic pain and who use tobacco may be at higher risk for non-medical opioid use if they are prescribed opioids, and should be encouraged to quit smoking and offered nicotine replacement therapy. Furthermore, stronger tobacco-control protections in rural areas may help combat opioid misuse in these regions.

## Figures and Tables

**Table 1:** Sample Characteristics.

Currently-Prescription Opioid	*n*	%(95% CI)
No	4098	85.20% (83.81%, 86.49%)
Yes	748	14.80% (13.51%, 16.19%)
*Tobacco use*		
No tobacco use	3608	72.81% (71.12%, 74.44%)
Traditional cigarette only	730	15.32% (13.94%, 16.80%)
E-cigarette only	87	1.81% (1.38%, 2.37%)
Cigar only	49	1.00% (0.70%, 1.42%)
Smokeless only	170	4.19% (3.50%, 5.02%)
Poly-tobacco product user	216	4.86% (4.16%, 5.67%)
*Race/ethnicity*		
NH White	4031	80.63% (75.52%, 84.89%)
NH African American	320	6.61% (4.61%, 9.39%)
NH Asian	29	0.72% (0.44%, 1.20%)
Latino	254	6.30% (3.98%, 9.85%)
NH Other	230	5.73% (3.23%, 9.96%)
*Age*		
18–34	876	24.37% (22.56%, 26.27%)
35–64	2263	48.92% (47.48%, 50.37%)
65 or older	1717	26.71% (25.14%, 28.34%)
*Sex*		
Male	2206	47.22% (45.39%, 49.60%)
Female	2658	52.78% (50.94%, 54.61%)
*Education*		
Not graduated from High School (HS)	611	16.13% (14.38%, 18.05%)
HS or GED	1671	34.26% (32.37%, 36.20%)
Some college	1518	32.45% (30.45%, 34.52%)
Bachelors or higher	1040	17.14% (15.37%, 19.06%)
*Income to federal poverty level ratio*		
Under 1	706	14.34% (12.54%, 16.34%)
1–1.99	1148	23.49% (21.97%, 25.09%)
2–3.99	1621	34.01% (32.34%, 35.73%)
4 or higher	1389	28.16% (25.55%, 30.92%)
*Region*		
Northeast	431	10.31% (8.41%, 12.59%)
Midwest	1722	32.96% (29.53%, 36.58%)
South	1964	42.34% (38.51%, 46.26%)
West	747	14.39% (11.06%, 18.51%)
N	5081	

**Table 2:** Association between tobacco use and medical opioid use.

	OR (95% CI)
*Tobacco use*	
No tobacco use	1
Traditional cigarette only	**1.62 (1.31, 2.01)**
E-cigarette only	0.92 (0.5, 1.67)
Cigar only	1.12 (0.34, 3.74)
Smokeless only	1.67 (0.99, 2.8)
Poly-tobacco product user	**2.13 (1.4, 3.22)**
*Race/ethnicity*	
NH White	1
NH African American	1.07 (0.76, 1.49)
NH Asian	0.25 (0.03, 2.03)
Latino	**0.53 (0.32, 0.86)**
NH Other	0.93 (0.47, 1.86)
*Age*	
18–34	**0.54 (0.4, 0.72)**
35–64	0.9 (0.73, 1.12)
65 or older	1
*Sex*	
Female	**1.91 (1.55, 2.34)**
Male	1
*Education*	
Below HS education	1 (0.7, 1.43)
HS or GED	1.03 (0.8, 1.33)
Some college	1.08 (0.81, 1.43)
Bachelors or higher	1
*Income to federal poverty level ratio*	
Under 1	**1.62 (1.18, 2.23)**
1–1.99	1.28 (0.93, 1.78)
2–3.99	1.3 (0.99, 1.7)
4 or higher	1
*Region*	
Northeast	0.98 (0.59, 1.62)
Midwest	1.15 (0.78, 1.69)
South	1.42 (0.96, 2.08)
West	1
Constant	**0.08 (0.05, 0.13)**
N	5028

## Data Availability

The data in this analysis is the publicly available sample adult file 2019 National Health Interview Survey (NHIS) can be downloaded from the CDC NHIS website.

## References

[1] Paulozzi L.J., Xi Y. (2008). Recent changes in drug poisoning mortality in the United States by urban-rural status and by drug type. Pharmacoepidemiol. Drug Saf..

[2] Garcia M.C., Rossen L.M., Bastian B., Faul M., Dowling N.F., Thomas C.C., Schieb L., Hong Y., Yoon P.W., Iademarco M.F. (2019). Potentially Excess Deaths from the Five Leading Causes of Death in Metropolitan and Nonmetropolitan Counties—United States, 2010–2017. MMWR Surveill. Summ..

[3] García M.C., Heilig C.M., Lee S.H., Faul M., Guy G., Iademarco M.F., Hempstead K., Raymond D., Gray J. (2019). Opioid Prescribing Rates in Nonmetropolitan and Metropolitan Counties Among Primary Care Providers Using an Electronic Health Record System—United States, 2014–2017. MMWR Morb. Mortal. Wkly. Rep..

[4] White A.G., Birnbaum H.G., Schiller M., Tang J., Katz N.P. (2009). Analytic models to identify patients at risk for prescription opioid abuse. Am. J. Manag. Care.

[5] Compton W.M., Boyle M., Wargo E. (2015). Prescription opioid abuse: Problems and responses. Prev. Med..

[6] Cicero T.J., Surratt H., Inciardi J.A., Munoz A. (2007). Relationship between therapeutic use and abuse of opioid analgesics in rural, suburban, and urban locations in the United States. Pharmacoepidemiol. Drug Saf..

[7] Erwin P.C. (2017). Despair in the American Heartland? A Focus on Rural Health. Am. J. Public Health.

[8] Stein E.M., Gennuso K.P., Ugboaja D.C., Remington P.L. (2017). The Epidemic of Despair Among White Americans: Trends in the Leading Causes of Premature Death, 1999–2015. Am. J. Public Health.

[9] George D.R., Snyder B., Van Scoy L.J., Brignone E., Sinoway L., Sauder C., Murray A., Gladden R., Ramedani S., Ernharth A. (2021). Perceptions of Diseases of Despair by Members of Rural and Urban High-Prevalence Communities: A Qualitative Study. JAMA Netw. Open.

[10] Kalman D., Morissette S.B., George T.P. (2005). Co-Morbidity of Smoking in Patients with Psychiatric and Substance Use Disorders. Am. J. Addict..

[11] Morris C.D., Garver-Apgar C.E. (2020). Nicotine and Opioids: A Call for Co-treatment as the Standard of Care. J. Behav. Health Serv. Res..

[12] LaRowe L.R., Ditre J.W. (2020). Pain, nicotine, and tobacco smoking: Current state of the science. Pain.

[13] Huxtable C.A., Roberts L.J., Somogyi A.A., MacIntyre P.E. (2011). Acute pain management in opioid-tolerant patients: A growing chal-lenge. Anaesth Intensive Care..

[14] Fishbain D.A., Cole B., Lewis J.E., Gao J. (2012). Is Smoking Associated with Alcohol-Drug Dependence in Patients with Pain and Chronic Pain Patients? An Evidence-Based Structured Review. Pain Med..

[15] Michna E., Ross E.L., Hynes W.L., Nedeljkovic S.S., Soumekh S., Janfaza D., Palombi D., Jamison R.N. (2004). Predicting aberrant drug behavior in patients treated for chronic pain: Importance of abuse history. J. Pain Symptom Manag..

[16] National Center for Health Statistics, Center for Disease Control and Prevention (2020). Survey Description, National Health Interview Survey, 2019.

[17] Center for Disease Control and Prevention Rural Health Policy Brief Preventing Opioid Overdoses in Rural America. https://www.cdc.gov/ruralhealth/drug-overdose/pdf/policy-brief_opioiod-overdoses-h.pdf.

[18] Roberts M.E., Berman M.L., Slater M.D., Hinton A., Ferketich A.K. (2015). Point-of-sale tobacco marketing in rural and urban Ohio: Could the new landscape of Tobacco products widen inequalities?. Prev. Med..

[19] Center for Disease Control and Prevention Tobacco Use by Geographic Region. https://www.cdc.gov/tobacco/disparities/geographic/index.htm.

[20] Brown-Johnson C.G., England L.J., Glantz S.A., Ling P.M. (2014). Tobacco industry marketing to low socioeconomic status women in the USA. Tob. Control.

[21] Buettner-Schmidt K., Miller D.R., Maack B. (2019). Disparities in Rural Tobacco Use, Smoke-Free Policies, and Tobacco Taxes. West. J. Nurs. Res..

[22] Coffin P.O., Rowe C., Oman N., Sinchek K., Santos G.-M., Faul M., Bagnulo R., Mohamed D., Vittinghoff E. (2020). Illicit opioid use following changes in opioids prescribed for chronic non-cancer pain. PLoS ONE.

[23] Newman A.K., Van Dyke B.P., Torres C.A., Baxter J.W., Eyer J.C., Kapoor S., Thorn B.E. (2017). The relationship of sociodemographic and psychological variables with chronic pain variables in a low-income population. Pain.

[24] Mazure C.M., Fiellin D.A. (2018). Women and opioids: Something different is happening here. Lancet.

[25] Chua K.-P., Hu H.-M., Waljee J.F., Brummett C.M., Nalliah R.P. (2021). Opioid prescribing patterns by dental procedure among US publicly and privately insured patients, 2013 through 2018. J. Am. Dent. Assoc..

[26] Karamouzian M., Papamihali K., Graham B., Crabtree A., Mill C., Kuo M., Young S., Buxton J.A. (2020). Known fentanyl use among clients of harm reduction sites in British Columbia, Canada. Int. J. Drug Policy.

